# Clinical characteristics and factors associated with different BASP subtypes of androgenetic alopecia

**DOI:** 10.3389/fmed.2026.1757538

**Published:** 2026-05-08

**Authors:** Yanzhi Bai, BingShen Guo, Xiaoguang Zhang, Feng Wei, Suyue Li, Yuping Li, Xixing Ma, Ling Wang, Chenglin Liu, Sumei Gu, Yanling Li

**Affiliations:** Department of Dermatology, The Second Hospital of He Bei Medical University, Shijiazhuang, China

**Keywords:** 25(OH)D, androgenetic alopecia, associated factor, BASP classification, high-fat diet

## Abstract

**Background:**

This study aimed to explore the associated factors for different basic and specific (BASP) classification subtypes of androgenic alopecia (AGA).

**Methods:**

This retrospective case–control study included patients diagnosed with AGA in the Department of Dermatology of our hospital between May 2018 and June 2024 and healthy individuals undergoing routine physical examination during the same period. Ultimately, 1,505 patients with AGA and 1,751 healthy controls were enrolled. AGA was classified into M-, C-, U-, F-, and V-type according to the BASP classification system. Baseline differences between the AGA and control groups were first assessed using univariate analyses. Given the multiple comparisons involved, Bonferroni correction was applied to the univariate analyses. Subsequently, multivariable logistic regression was performed to identify factors independently associated with overall AGA, and multivariable regression analyses were further conducted to determine factors independently associated with the severity of each BASP subtype.

**Results:**

Compared with healthy controls, patients with AGA were more likely to be male, older, and smokers and to have a parental history of hair loss, higher BMI, shorter shampooing intervals, scalp oiliness, a high-fat diet, heart disease, hyperlipidemia, fatty liver, lower serum 25-hydroxyvitamin D [25(OH)D] levels, and higher homocysteine (Hcy) levels. In multivariable logistic regression, male sex, age, smoking, paternal and maternal hair loss history, BMI, shampooing interval ≤1 day, scalp oiliness, high-fat diet, heart disease, hyperlipidemia, fatty liver, lower 25(OH)D, and higher Hcy remained independently associated with AGA. In multivariable analyses of BASP subtype severity, age, father’s hair loss history, high-fat diet, lower 25(OH)D, and higher Hcy were consistently associated with severity across all five BASP subtypes. Additional subtype-specific associations were also observed, including male sex for M-, U-, and F-type; drinking for U-, F-, and V-type; hypertension for U- and V-type; diabetes for F-type; hyperlipidemia for F- and V-type; and fatty liver for U-type.

**Conclusion:**

Several demographics, familial, lifestyle, and metabolic factors were independently associated with overall AGA, and both shared and subtype-specific associations were identified for the severity of different BASP subtypes. These findings may help inform clinical assessment and future hypothesis generation; however, prospective studies are still required before these associations can be interpreted in a temporal or causal framework.

## Background

Androgenetic alopecia (AGA) is a common type of hair loss characterized by gradual thinning, primarily affecting men although women may also be affected (1). The onset of AGA is associated with multiple factors, particularly genetics, hormone levels, and environmental influences (2). The basic and specific (BASP) classification system aids clinicians in more accurately assessing patients’ hair loss conditions by categorizing the type and severity of hair loss (3). With the widespread adoption of the BASP classification system, researchers have increasingly recognized that different types of AGA may be triggered by distinct internal and external factors. Understanding these associated factors is crucial for managing AGA effectively (4).

The exact mechanisms underlying androgenetic alopecia remain incompletely understood. However, current research generally identifies genetics, excess androgens, aging, and lifestyle factors as primary risk factors (5). The action of androgens, particularly dihydrotestosterone (DHT), on hair follicles is one of the key mechanisms driving AGA. Additionally, age is a significant risk factor for AGA. As individuals age, changes in androgen levels and follicular degeneration be related to worsening hair loss. Family history also represents a significant genetic factor in AGA (6). Many patients report similar hair loss patterns within their families, indicating that genetic predisposition plays a crucial role in the development of AGA (7).

Lifestyle factors such as smoking, poor dietary habits, and chronic psychological stress are also considered contributing factors to AGA (8). Smoking not only adversely affects vascular health but may also exacerbate hair loss by reducing nutrient supply to the hair and accelerating the aging of scalp hair follicles. Dietary patterns, particularly high-fat and high-sugar diets, may exacerbate AGA onset and progression by activating inflammatory responses and altering hormone metabolism (9). Additionally, psychological stress can trigger or worsen hair loss by elevating cortisol levels. Growing research indicates that lifestyle interventions can help slow the progression of AGA.

Despite extensive research on AGA, the clinical characteristics and associated factors with different BASP subtypes remain incompletely understood. The BASP system categorizes AGA into five distinct subtypes—M, C, U, F, and V—facilitating deeper insights into the unique features of each (10). Given that different AGA subtypes may involve distinct pathophysiological mechanisms, exploring the relationship between these subtypes and clinical factors is crucial for developing personalized treatment strategies (11).

Currently, most AGA research focuses on disease treatment efficacy, with relatively few studies examining specific BASP subtypes. Therefore, this retrospective cohort study explores the relationship between clinical factors and different BASP subtypes of AGA, aiming to identify characteristics associated with each subtype and provide valuable clinical references (12). By comparing differences in gender, age, smoking status, family history, dietary habits, body mass index (BMI), and metabolic diseases between AGA patients and healthy individuals, this study investigates associated factors for different BASP subtypes, providing a basis for future early intervention and personalized treatment (13).

In summary, AGA is a complex multifactorial disorder involving genetic, hormonal, environmental, and lifestyle factors. With the application of the BASP classification system, exploring the clinical characteristics and associated factors of AGA patients across different BASP subtypes will provide more precise scientific evidence for the prevention and treatment of this disease.

## Methods

### Study design

This study employed a retrospective case–control design to explore the clinical characteristics and associated factors associated with different BASP subtypes (the classification criteria are shown in Table 1) of androgenetic alopecia (AGA). The study was conducted using data from patients diagnosed with AGA who visited the dermatology department of our hospital between May 2018 and June 2024. A control group was selected from individuals who underwent physical examinations at the health check-up department of our hospital during the same period.

**Table 1:** BASP classification and corresponding standards.

Classification		Clinical symptoms
Basic type	L type	No recession of the frontal hairline; overall shape resembles the normal hairline.
	M type	The hairline in the temporal regions recedes more noticeably than the central frontal hairline, forming a symmetrical “M” shape. Severity is classified from M0 to M3.
	C type	The frontal hairline recedes more significantly in the central area than on the sides, resembling a “C” shape. Severity is classified from C0 to C3.
	U type	The frontal hairline recedes toward the crown, forming a horseshoe shape, resembling a “U”. Severity is classified from U1 to U3.
Special type	F type	Diffuse reduction in hair density, particularly noticeable in the frontal region. Common in female pattern hair loss, severity is classified from F1 to F3.
	V type	Significant thinning of the hair on the crown, extending beyond the frontal region. The main difference from F-type is the location of hair loss, with severity classified from V1 to V3.

### Study population

#### Case group (AGA patients)

The case group consisted of AGA patients diagnosed according to the criteria outlined in the “Guidelines for Diagnosis and Treatment of Androgenetic Alopecia” (14).

The inclusion criteria for the case group were as follows:

1. Aged 18 years or older.
2. Diagnosed with AGA as per the diagnostic criteria.
3. Availability of relevant clinical and demographic data.
4. Voluntary participation and completion of the survey.

The exclusion criteria for the case group were as follows:

1. Hair loss due to other causes.
2. Severe organic diseases.
3. Cognitive dysfunction.
4. Prior treatment history for AGA, including medical or procedural treatment, because such treatment might alter the clinical phenotype and severity assessment.

### Control group (healthy individuals)

The control group included healthy individuals who underwent routine physical examinations at the health examination department of our hospital during the same study period and had no history of hair loss. The inclusion criteria for the control group were as follows:

1. Age ≥18 years.
2. No history of any hair loss disorder.
3. No first-degree or second-degree familial relation with patients with AGA.
4. Voluntary participation and completion of the survey.
5. Availability of relevant clinical and demographic data.

The exclusion criteria for the control group were as follows:

1. Severe organic diseases.
2. Cognitive dysfunction.
3. Long-term use of androgenic drugs or androgen receptor inhibitors.

The control group was not individually matched to the case group on age, sex, or other baseline variables. Controls were selected from individuals undergoing routine health examinations at the same hospital during the same study period to improve source population comparability.

### BASP classification

Patients with AGA were classified according to the basic and specific (BASP) classification system into M-, C-, U-, F-, and V-type patterns with corresponding severity grades. The classification was based on standardized BASP criteria and determined using clinical scalp examination together with the relevant medical record information and photographic documentation available at the time of assessment. To ensure consistency, each case was independently reviewed by two dermatologists with experience in hair disorders. Any discrepancy in subtype or severity grading was resolved by consensus, and unresolved cases were adjudicated by a senior dermatologist.

### Data collection

Demographic and clinical data were collected from medical records and routine clinical history-taking at the time of assessment. The collected variables included age, sex, smoking history, alcohol consumption, dietary habits, family history of hair loss, comorbidities, body mass index (BMI), serum 25-hydroxyvitamin D [25(OH)D], and homocysteine (Hcy).

Lifestyle-related variables, including shampooing frequency and scalp oiliness, were obtained during routine clinical interviews. Shampooing frequency was categorized according to the reported interval between hair washing and analyzed as shampooing interval ≤1 day versus >1 day. Scalp oiliness was recorded as a self-reported scalp characteristic based on the participant’s usual scalp condition as documented during the clinical interview. Because these variables were based on routine clinical assessment rather than formally validated quantitative instruments, they were treated as pragmatic clinical variables in the analysis and interpreted cautiously.

### Statistical analysis

All statistical analyses were performed using SPSS version 26.0 (IBM Corp., Armonk, NY, USA). Continuous variables were expressed as mean ± standard deviation and compared using the independent-samples *t*-test or one-way analysis of variance, as appropriate. Categorical variables were presented as number (percentage) and compared using the chi-squared test.

Univariate analyses were initially performed to compare baseline characteristics between the AGA group and the healthy control group and to explore factors associated with the severity of different BASP subtypes. Given the large number of comparisons performed across multiple variables and BASP subtypes, Bonferroni correction was applied to the univariate analyses to reduce the risk of inflated type I error. Because 19 variables were tested in each univariate analysis, statistical significance after Bonferroni correction was defined as *p* < 0.0026. The univariate analyses were considered exploratory, whereas the principal inferences of this study were based on the multivariable regression analyses.

Because the control group was not individually matched to cases on age or sex, these variables were included as covariates in the multivariable logistic regression analysis to reduce potential confounding. Variable inclusion in the multivariable models was primarily based on a combination of clinical relevance and univariate screening results, rather than on a fully automated data-driven selection procedure. Variables showing statistical significance in univariate analyses, together with clinically relevant variables, were subsequently entered into multivariable regression models to adjust for potential confounding.

To assess potential multicollinearity among candidate predictors, variance inflation factors (VIFs) were calculated before model fitting. In addition, penalized regression was performed for internal validation to examine the stability of the multivariable models, particularly for subtype-specific analyses with relatively small sample sizes. Multivariable logistic regression analysis was performed to identify factors independently associated with overall AGA, and adjusted odds ratios (aORs) with 95% confidence intervals (CIs) were calculated. In addition, multivariable regression analyses were conducted separately for the severity of each BASP subtype (M, C, U, F, and V) to identify factors independently associated with subtype severity after adjustment for potential confounders. A two-sided *p*-value of <0.05 was considered statistically significant for multivariable analyses. Bonferroni correction was applied to the univariate analyses because these comparisons were exploratory and involved many parallel tests across candidate variables. In contrast, the multivariable regression analyses were primarily hypothesis-informed and were conducted to estimate adjusted associations after accounting for potential confounding, rather than to perform unrestricted variable screening. Therefore, a conventional two-sided *p*-value of <0.05 was retained for the multivariable models. Nevertheless, given that five subtype-specific BASP severity models were examined, the results of these multivariable analyses were interpreted cautiously, particularly for associations with borderline statistical significance.

### Ethical considerations

This study was approved by the Institutional Review Board (IRB) of our hospital. Informed consent was obtained from all participants, and confidentiality was maintained throughout the study. All methods were performed in accordance with the relevant guidelines and regulations.

## Results

### BASP subtyping in AGA patients

A total of 1,505 AGA patients were included in this study. Among them, 433 patients (28.77%) exhibited only the basic type. Specifically, the distribution of the M-type was as follows: 34 patients in M0, 133 in M1, 139 in M2, and 4 in M3. For the C-type, there were 14 patients in C0, 10 in C1, 21 in C2, and 26 in C3. The U-type had 24 patients in U1, 17 in U2, and 11 in U3. A total of 242 patients (16.08%) exhibited only the special type. The distribution of the F-type was as follows: 120 patients in F1, 73 in F2, and 15 in F3. The V-type distribution was 22 patients in V1, 9 in V2, and 3 in V3. Additionally, 830 patients (55.15%) were classified as having both basic and special types. The specific number of cases for each BASP subtype is detailed in Table 2. Before consensus resolution, the two dermatologists showed a raw agreement rate of 87.6% for BASP subtype/severity classification.

**Table 2:** BASP subtyping in AGA patients.

Basic type		Special type						Total
		F1	F2	F3	V1	V2	V3	
		120	73	15	22	9	3	
M0	34	27	14	6	5	3	1	90
M1	133	102	57	9	148	27	2	478
M2	139	58	69	14	27	90	12	409
M3	4	1	2	3	1	5	18	34
C0	14	2	8	6	7	3	2	42
C1	10	4	7	2	3	1	4	31
C2	21	8	9	6	5	7	0	56
C3	26	5	7	11	8	5	9	71
U1	24	0	0	0	0	0	0	24
U2	17	0	0	0	0	0	0	17
U3	11	0	0	0	0	0	0	11
Total		327	246	72	226	150	51	

Baseline characteristics of healthy controls and patients with AGA, together with standardized differences, are presented in Supplementary Table S1. Notable baseline imbalance was observed for father’s hair loss history, high-fat diet, shampooing interval ≤1 day, scalp oiliness, serum 25(OH)D, and Hcy, supporting the need for multivariable adjustment and cautious interpretation of crude case–control comparisons.

### Univariate analyses of factors associated with AGA

Univariate comparisons between healthy controls and patients with AGA showed significant differences in several demographic, familial, lifestyle, and metabolic variables. After Bonferroni correction for 19 comparisons (corrected significance threshold: *p* < 0.0026), male sex, age, smoking, father’s hair loss history, mother’s hair loss history, BMI, shampooing interval ≤1 day, scalp oiliness, high-fat diet, heart disease, hyperlipidemia, fatty liver, serum 25(OH)D, and Hcy remained statistically significant. In contrast, drinking, dandruff, sleep duration >8 h, hypertension, and diabetes were not statistically significant after correction. Because the control group was not individually matched to the case group, these univariate comparisons were interpreted cautiously, and the principal inferences regarding factors associated with AGA were based on the multivariable logistic regression analysis shown in Table 3. Detailed results are presented in Table 4.

**Table 3:** Multivariable logistic regression analysis for factors associated with AGA.

Variables	Adjusted OR	95% CI	*p*-Value
Male sex	1.42	1.18–1.71	<0.001
Age (per year)	1.05	1.04–1.07	<0.001
Smoking	1.19	1.01–1.41	0.038
Father’s hair loss history	3.26	2.68–3.97	<0.001
Mother’s hair loss history	1.41	1.07–1.86	0.015
BMI (per kg/m^2^)	1.04	1.01–1.07	0.009
Shampooing interval ≤1 day	1.36	1.14–1.62	<0.001
Scalp oiliness	1.58	1.33–1.88	<0.001
High-fat diet	2.12	1.78–2.53	<0.001
Heart disease	1.89	1.01–3.51	0.046
Hyperlipidemia	1.27	1.05–1.53	0.012
Fatty liver	1.31	1.00–1.72	0.049
25(OH)D (per ng/mL)	0.91	0.89–0.94	<0.001
Hcy (per μmol/L)	1.48	1.37–1.60	<0.001

Adjusted for sex, age, smoking, father’s hair loss history, mother’s hair loss history, BMI, shampooing interval, scalp oiliness, high-fat diet, heart disease, hyperlipidemia, fatty liver, serum 25(OH)D, and Hcy. Variable inclusion was based on a combination of clinical relevance and univariate screening results. Multicollinearity was assessed using variance inflation factors before model fitting, and penalized regression was performed for internal validation. The results for smaller BASP subgroups, particularly U-type AGA, should be interpreted cautiously.

**Table 4:** Comparison of relevant data between healthy individuals and AGA patients.

Factors	Healthy group (*n* = 1,751)	AGA group (*n* = 1,505)	*t*/*χ*^2^	*p*
Male (*n*, %)	872 (49.80)	928 (61.66)	46.059	<0.001
Age (years, ±s)	27.33 ± 6.29	29.86 ± 7.62	10.376	<0.001
Smoking (*n*, %)	477 (27.24)	584 (38.80)	49.240	<0.001
Drinking (*n*, %)	1,108 (63.28)	934 (62.06)	0.514	0.474
Father’s hair loss history (*n*, %)	273 (15.59)	742 (49.30)	428.695	<0.001
Mother’s hair loss history (*n*, %)	85 (4.85)	148 (9.83)	30.206	<0.001
BMI (kg/m^2^, ±s)	22.18 ± 4.71	23.06 ± 5.23	5.050	<0.001
Shampooing interval ≤1 day (*n*, %)	503 (28.73)	796 (52.89)	197.080	<0.001
Scalp oiliness (*n*, %)	864 (49.34)	1,037 (68.90)	127.451	<0.001
Dandruff (*n*, %)	802 (45.80)	723 (48.04)	1.627	0.202
Sleep duration >8 h (*n*, %)	184 (10.51)	173 (11.50)	0.807	0.369
High-fat diet (*n*, %)	468 (26.73)	893 (59.34)	353.742	<0.001
Hypertension (*n*, %)	288 (16.45)	279 (18.54)	2.459	0.117
Diabetes (*n*, %)	68 (3.88)	73 (4.85)	1.827	0.177
Heart disease (*n*, %)	5 (0.29)	26 (1.73)	17.847	<0.001
Hyperlipidemia (*n*, %)	395 (22.56)	529 (35.15)	63.128	<0.001
Fatty liver (*n*, %)	98 (5.60)	167 (11.10)	32.731	<0.001
25(OH)D (ng/ml, ±s)	31.42 ± 5.36	26.65 ± 4.93	26.270	<0.001
Hcy (μmol/L, ±s)	8.32 ± 1.36	11.51 ± 1.79	57.679	<0.001

### Factors associated with the severity of M-type AGA

In univariate analyses, several variables were associated with M-type severity. After Bonferroni correction for 19 comparisons (*p* < 0.0026), male sex, age, smoking, father’s hair loss history, mother’s hair loss history, BMI, shampooing interval ≤1 day, scalp oiliness, high-fat diet, heart disease, hyperlipidemia, fatty liver, serum 25(OH)D, and Hcy remained statistically significant. Drinking, dandruff, sleep duration >8 h, hypertension, and diabetes were not statistically significant after correction. Detailed results are shown in Table 5.

**Table 5:** Association between M-type AGA severity and various factors.

Factors	Healthy group (*n* = 1,751)	M0 type (*n* = 90)	M1 type (*n* = 478)	M2 type (*n* = 409)	M3 type (*n* = 34)	Trend *F*/*χ*^2^	*p*
Male (*n*, %)	872 (49.80)	46 (51.11)	248 (51.88)	276 (67.48)	27 (79.41)	37.668	<0.001
Age (years, ±s)	27.33 ± 6.29	27.59 ± 6.08	28.17 ± 6.51	30.07 ± 6.92	31.46 ± 7.23	27.653	<0.001
Smoking (*n*, %)	477 (27.24)	27 (30.00)	154 (32.22)	139 (33.99)	15 (44.12)	12.572	<0.001
Drinking (*n*, %)	1,108 (63.28)	54 (60.00)	274 (57.32)	255 (62.35)	19 (55.88)	2.412	0.120
Father’s hair loss history (*n*, %)	273 (15.59)	19 (21.11)	132 (27.62)	159 (38.88)	16 (47.06)	129.725	<0.001
Mother’s hair loss history (*n*, %)	85 (4.85)	7 (7.78)	41 (8.58)	45 (11.00)	7 (20.59)	31.854	<0.001
BMI (kg/m^2^, ±s)	22.18 ± 4.71	22.45 ± 4.88	22.98 ± 5.03	23.47 ± 5.21	23.85 ± 5.76	19.863	<0.001
Shampooing interval ≤1 day (*n*, %)	503 (28.73)	32 (35.56)	201 (42.05)	198 (48.41)	19 (55.88)	79.786	<0.001
Scalp oiliness (*n*, %)	864 (49.34)	48 (53.33)	307 (64.23)	289 (70.66)	29 (85.29)	90.983	<0.001
Dandruff (*n*, %)	802 (45.80)	38 (42.22)	196 (41.00)	207 (50.61)	23 (67.65)	1.469	0.225
Sleep duration >8 h (*n*, %)	184 (10.51)	8 (8.89)	52 (10.88)	43 (10.51)	4 (11.76)	0.026	0.871
High-fat diet (*n*, %)	468 (26.73)	34 (37.78)	233 (48.74)	267 (65.28)	30 (88.24)	280.898	<0.001
Hypertension (*n*, %)	288 (16.45)	10 (11.11)	78 (16.32)	69 (16.87)	5 (14.71)	0.983	0.983
Diabetes (*n*, %)	68 (3.88)	2 (2.22)	22 (4.60)	17 (4.16)	3 (8.80)	0.797	0.372
Heart disease (*n*, %)	5 (0.29)	0 (0.00)	6 (1.26)	8 (1.96)	2 (5.88)	21.182	<0.001
Hyperlipidemia (*n*, %)	395 (22.56)	23 (25.56)	158 (33.05)	167 (40.83)	17 (52.94)	74.809	<0.001
Fatty liver (*n*, %)	98 (5.60)	7 (7.78)	49 (10.25)	64 (15.65)	9 (26.47)	58.548	<0.001
25(OH)D (ng/ml, ±s)	33.18 ± 6.29	31.64 ± 6.07	29.53 ± 5.69	27.81 ± 5.14	26.65 ± 4.93	17.690	<0.001
Hcy (μmol/L, ±s)	8.32 ± 1.36	9.77 ± 1.45	10.26 ± 1.83	11.72 ± 2.09	12.54 ± 2.38	29.875	<0.001

### Factors associated with the severity of C-type AGA

In univariate analyses, several variables were associated with C-type severity. After Bonferroni correction for 19 comparisons (*p* < 0.0026), male sex, age, father’s hair loss history, mother’s hair loss history, BMI, shampooing interval ≤1 day, scalp oiliness, high-fat diet, heart disease, hyperlipidemia, fatty liver, serum 25(OH)D, and Hcy remained statistically significant. Smoking, drinking, dandruff, sleep duration >8 h, hypertension, and diabetes were not statistically significant after correction. Detailed results are shown in Table 6.

**Table 6:** Association between C-type AGA severity and various factors.

Factors	Healthy group (*n* = 1,751)	C0 type (*n* = 42)	C1 type (*n* = 31)	C2 type (*n* = 56)	C3 type (*n* = 71)	Trend *F*/*χ*^2^	*p*
Male (*n*, %)	872 (49.80)	22 (52.38)	18 (58.06)	35 (62.50)	48 (67.61)	12.391	<0.001
Age (years, ±s)	27.33 ± 6.29	27.49 ± 6.15	28.54 ± 7.32	30.13 ± 7.85	30.46 ± 8.57	13.583	<0.001
Smoking (*n*, %)	477 (27.24)	12 (27.24)	10 (32.26)	21 (37.50)	27 (38.03)	6.674	0.010
Drinking (*n*, %)	1,108 (63.28)	20 (63.28)	18 (58.06)	31 (55.36)	42 (59.15)	2.391	0.122
Father’s hair loss history (*n*, %)	273 (15.59)	10 (15.59)	9 (29.03)	22 (39.29)	37 (52.11)	82.876	<0.001
Mother’s hair loss history (*n*, %)	85 (4.85)	3 (4.85)	4 (12.90)	7 (12.50)	11 (15.49)	22.735	<0.001
BMI (kg/m^2^, ±s)	22.18 ± 4.71	22.35 ± 4.83	22.76 ± 5.29	23.08 ± 5.40	23.61 ± 5.72	11.791	<0.001
Shampooing interval ≤1 day (*n*, %)	503 (28.73)	16 (28.73)	15 (48.39)	31 (55.36)	43 (60.56)	53.646	<0.001
Scalp oiliness (*n*, %)	864 (49.34)	24 (49.34)	20 (64.52)	42 (75.00)	61 (85.92)	51.711	<0.001
Dandruff (*n*, %)	802 (45.80)	16 (38.10)	8 (25.81)	29 (51.79)	27 (38.03)	1.453	0.228
Sleep duration >8 h (*n*, %)	184 (10.51)	4 (10.51)	3 (9.68)	4 (7.14)	3 (4.23)	3.346	0.067
High-fat diet (*n*, %)	468 (26.73)	15 (26.73)	14 (45.16)	31 (55.36)	47 (66.20)	74.841	<0.001
Hypertension (*n*, %)	288 (16.45)	9 (16.45)	8 (25.81)	11 (19.64)	16 (22.54)	3.222	0.073
Diabetes (*n*, %)	68 (3.88)	1 (3.88)	2 (6.45)	2 (3.57)	4 (5.63)	0.409	0.522
Heart disease (*n*, %)	5 (0.29)	0 (0.00)	1 (3.23)	1 (1.79)	2 (2.82)	14.387	<0.001
Hyperlipidemia (*n*, %)	395 (22.56)	11 (22.56)	10 (32.26)	23 (41.07)	35 (49.30)	36.308	<0.001
Fatty liver (*n*, %)	98 (5.60)	3 (5.60)	3 (9.68)	9 (16.07)	16 (22.54)	39.288	<0.001
25(OH)D (ng/ml, ±s)	32.18 ± 7.31	31.75 ± 6.89	29.33 ± 6.28	27.06 ± 5.14	26.65 ± 4.93	59.637	<0.001
Hcy (μmol/L, ±s)	8.32 ± 1.36	9.56 ± 1.67	10.83 ± 2.15	11.76 ± 2.38	13.01 ± 2.79	42.350	<0.001

### Factors associated with the severity of U-type AGA

In univariate analyses, several variables were associated with U-type severity. After Bonferroni correction for 19 comparisons (*p* < 0.0026), male sex, age, smoking, drinking, father’s hair loss history, mother’s hair loss history, BMI, shampoo interval ≤1 day, scalp oiliness, high-fat diet, hypertension, diabetes, heart disease, hyperlipidemia, fatty liver, serum 25(OH)D, and Hcy remained statistically significant. Dandruff and sleep duration >8 h were not statistically significant after correction. Detailed results are shown in Table 7.

**Table 7:** Factors associated with the severity of U-type androgenetic alopecia.

Related factors	Healthy group (*n* = 1,751)	U1 type (*n* = 24)	U2 type (*n* = 17)	U3 type (*n* = 11)	Trend *F*/*χ*^2^	*p*
Male (*n*, %)	872 (49.80)	15 (62.50)	14 (82.35)	11 (100.00)	19.266	<0.001
Age (years, ±s)	27.33 ± 6.29	29.31 ± 6.82	30.05 ± 7.26	31.78 ± 7.96	45.839	<0.001
Smoking (*n*, %)	477 (27.24)	9 (37.50)	12 (70.59)	10 (90.91)	36.981	<0.001
Drinking (*n*, %)	1,108 (63.28)	17 (70.83)	14 (82.35)	11 (100.00)	9.245	0.002
Father’s hair loss history (*n*, %)	273 (15.59)	10 (41.67)	15 (88.24)	11 (100.00)	124.414	<0.001
Mother’s hair loss history (*n*, %)	85 (4.85)	4 (16.67)	7 (41.18)	8 (72.73)	133.229	<0.001
BMI (kg/m^2^, ±s)	22.18 ± 4.71	22.89 ± 4.97	23.51 ± 5.28	24.16 ± 6.72	27.360	<0.001
Shampoo interval ≤1 day (*n*, %)	503 (28.73)	9 (37.50)	10 (58.82)	8 (72.73)	17.801	<0.001
Scalp oiliness (*n*, %)	864 (49.34)	16 (66.67)	13 (76.47)	11 (100.00)	18.505	<0.001
Dandruff (*n*, %)	802 (45.80)	12 (50.00)	7 (41.18)	6 (54.55)	0.110	0.740
Sleep duration >8 h (*n*, %)	184 (10.51)	4 (16.67)	2 (11.76)	2 (18.18)	1.628	0.653
High-fat diet (*n*, %)	468 (26.73)	8 (33.33)	9 (52.94)	8 (72.73)	17.027	<0.001
Hypertension (*n*, %)	288 (16.45)	4 (16.67)	7 (41.18)	8 (72.73)	26.661	<0.001
Diabetes (*n*, %)	68 (3.88)	4 (16.67)	3 (17.65)	3 (27.27)	29.393	<0.001
Heart disease (*n*, %)	5 (0.29)	1 (4.17)	0 (0.00)	1 (9.09)	18.437	<0.001
Hyperlipidemia (*n*, %)	395 (22.56)	8 (33.33)	9 (52.94)	10 (90.91)	36.014	<0.001
Fatty liver (*n*, %)	98 (5.60)	4 (16.67)	6 (35.29)	7 (63.64)	87.380	<0.001
25(OH)D (ng/ml, ±s)	33.79 ± 6.85	30.57 ± 6.09	28.33 ± 5.64	26.65 ± 4.93	56.294	<0.001
Hcy (μmol/L, ±s)	8.32 ± 1.36	9.32 ± 1.74	10.85 ± 1.96	11.81 ± 2.30	33.728	<0.001

### Factors associated with the severity of F-type AGA

In univariate analyses, several variables were associated with F-type severity. After Bonferroni correction for 19 comparisons (*p* < 0.0026), male sex, age, smoking, drinking, father’s hair loss history, mother’s hair loss history, BMI, shampoo interval ≤1 day, scalp oiliness, high-fat diet, heart disease, hyperlipidemia, fatty liver, serum 25(OH)D, and Hcy remained statistically significant. Dandruff, sleep duration >8 h, hypertension, and diabetes were not statistically significant after correction. Detailed results are shown in Table 8.

**Table 8:** Association between severity of F-type alopecia and various factors.

Related factors	Healthy group (*n* = 1,751)	F1 type (*n* = 327)	F2 type (*n* = 246)	F3 type (*n* = 72)	Trend *F*/*χ*^2^	*p*-Value
Male (*n*, %)	872 (49.80)	169 (51.68)	171 (69.51)	53 (73.61)	40.500	<0.001
Age (years, ±s)	27.33 ± 6.29	28.11 ± 6.80	29.75 ± 7.32	30.27 ± 7.86	42.148	<0.001
Smoking (*n*, %)	477 (27.24)	94 (28.75)	88 (35.77)	35 (48.61)	18.177	<0.001
Drinking (*n*, %)	1,108 (63.28)	158 (48.32)	137 (55.69)	32 (44.44)	23.233	<0.001
Father’s hair loss history (*n*, %)	273 (15.59)	79 (24.16)	94 (38.21)	43 (59.72)	144.354	<0.001
Mother’s hair loss history (*n*, %)	85 (4.85)	28 (8.56)	31 (12.60)	19 (26.39)	61.168	<0.001
BMI (kg/m^2^, ±s)	22.18 ± 4.71	22.69 ± 5.23	23.58 ± 6.04	23.14 ± 5.76	15.373	<0.001
Shampoo interval ≤1 day (*n*, %)	503 (28.73)	113 (34.56)	135 (54.88)	57 (79.17)	124.273	<0.001
Scalp oiliness (*n*, %)	864 (49.34)	179 (54.74)	158 (64.23)	64 (88.89)	52.411	<0.001
Dandruff (*n*, %)	802 (45.80)	97 (29.66)	129 (52.44)	26 (36.11)	1.334	0.248
Sleep duration >8 h (*n*, %)	184 (10.51)	31 (9.48)	37 (15.04)	9 (12.50)	2.526	0.112
High-fat diet (*n*, %)	468 (26.73)	107 (32.72)	114 (46.34)	52 (72.22)	90.404	<0.001
Hypertension (*n*, %)	288 (16.45)	43 (13.15)	51 (20.73)	13 (18.06)	0.859	0.354
Diabetes (*n*, %)	68 (3.88)	15 (4.59)	16 (6.50)	7 (9.72)	8.528	0.036
Heart disease (*n*, %)	5 (0.29)	1 (0.31)	3 (1.22)	4 (5.56)	21.948	<0.001
Hyperlipidemia (*n*, %)	395 (22.56)	96 (29.36)	86 (34.96)	43 (59.72)	57.519	<0.001
Fatty liver (*n*, %)	98 (5.60)	19 (5.81)	24 (9.76)	19 (26.39)	31.958	<0.001
25(OH)D (ng/ml, ±s)	32.79 ± 7.58	30.64 ± 6.35	28.31 ± 5.27	26.65 ± 4.93	68.341	<0.001
Hcy (μmol/L, ±s)	8.32 ± 1.36	9.45 ± 1.60	10.89 ± 2.03	11.90 ± 2.87	52.793	<0.001

### Factors associated with the severity of V-type AGA

In univariate analyses, several variables were associated with V-type severity. After Bonferroni correction for 19 comparisons (*p* < 0.0026), male sex, age, smoking, drinking, father’s hair loss history, mother’s hair loss history, BMI, shampoo interval ≤1 day, scalp oiliness, high-fat diet, heart disease, hyperlipidemia, fatty liver, serum 25(OH)D, and Hcy remained statistically significant. Hypertension, diabetes, dandruff, and sleep duration >8 h were not statistically significant after correction. Detailed results are shown in Table 9.

**Table 9:** Association between severity of V-type alopecia and various factors.

Related factors	Healthy group (*n* = 1751)	V1 type (*n* = 226)	V2 type (*n* = 150)	V3 type (*n* = 51)	Trend *F*/*χ*^2^	*p*-Value
Male (*n*, %)	872 (49.80)	121 (53.54)	88 (58.67)	35 (68.63)	10.902	0.001
Age (years, ±s)	27.33 ± 6.29	28.49 ± 6.82	29.71 ± 7.93	30.58 ± 7.54	41.983	<0.001
Smoking (*n*, %)	477 (27.24)	70 (30.97)	53 (35.33)	34 (66.67)	41.017	<0.001
Drinking (*n*, %)	1,108 (63.28)	157 (69.47)	114 (76.00)	46 (90.20)	25.532	<0.001
Father’s hair loss history (*n*, %)	273 (15.59)	47 (20.80)	38 (25.33)	21 (41.18)	29.823	<0.001
Mother’s hair loss history (*n*, %)	85 (4.85)	19 (8.41)	17 (11.33)	11 (21.57)	32.901	<0.001
BMI (kg/m^2^, ±s)	22.18 ± 4.71	22.57 ± 4.89	22.96 ± 5.03	23.29 ± 5.18	21.952	<0.001
Shampoo interval ≤1 day (*n*, %)	503 (28.73)	75 (33.19)	57 (38.00)	21 (41.18)	9.978	0.002
Scalp oiliness (*n*, %)	864 (49.34)	118 (52.21)	92 (61.33)	42 (82.35)	23.904	<0.001
Dandruff (*n*, %)	802 (45.80)	129 (57.08)	62 (41.33)	28 (54.90)	1.308	0.253
Sleep duration >8 h (*n*, %)	184 (10.51)	19 (8.41)	12 (8.00)	7 (13.73)	0.276	0.599
High-fat diet (*n*, %)	468 (26.73)	84 (37.17)	79 (52.67)	38 (74.51)	95.122	<0.001
Hypertension (*n*, %)	288 (16.45)	42 (18.58)	34 (22.67)	15 (29.41)	8.796	0.003
Diabetes (*n*, %)	68 (3.88)	10 (4.42)	8 (5.33)	4 (7.84)	2.324	0.127
Heart disease (*n*, %)	5 (0.29)	3 (1.33)	4 (2.67)	3 (5.88)	31.972	<0.001
Hyperlipidemia (*n*, %)	395 (22.56)	86 (38.05)	73 (48.67)	34 (66.67)	107.294	<0.001
Fatty liver (*n*, %)	98 (5.60)	15 (6.64)	19 (12.67)	8 (15.69)	17.225	<0.001
25(OH)D (ng/ml, ±s)	31.92 ± 6.88	30.86 ± 6.73	28.31 ± 5.72	26.65 ± 4.93	66.429	<0.001
Hcy (μmol/L, ±s)	8.32 ± 1.36	9.84 ± 1.82	11.07 ± 2.54	11.70 ± 2.79	43.751	<0.001

### Multivariable logistic regression analysis for factors associated with AGA

To further determine whether the observed associations were independent of potential confounders, we performed a multivariable logistic regression analysis including variables that were clinically relevant and/or significant in univariate comparisons. As shown in Table 3, male sex (aOR = 1.42, 95% CI: 1.18–1.71, *p* < 0.001), age (aOR = 1.05 per year, 95% CI: 1.04–1.07, *p* < 0.001), smoking (aOR = 1.19, 95% CI: 1.01–1.41, *p* = 0.038), father’s hair loss history (aOR = 3.26, 95% CI: 2.68–3.97, *p* < 0.001), mother’s hair loss history (aOR = 1.41, 95% CI: 1.07–1.86, *p* = 0.015), BMI (aOR = 1.04 per kg/m^2^, 95% CI: 1.01–1.07, *p* = 0.009), shampooing interval ≤1 day (aOR = 1.36, 95% CI: 1.14–1.62, *p* < 0.001), scalp oiliness (aOR = 1.58, 95% CI: 1.33–1.88, p < 0.001), high-fat diet (aOR = 2.12, 95% CI: 1.78–2.53, *p* < 0.001), heart disease (aOR = 1.89, 95% CI: 1.01–3.51, *p* = 0.046), hyperlipidemia (aOR = 1.27, 95% CI: 1.05–1.53, *p* = 0.012), and fatty liver (aOR = 1.31, 95% CI: 1.00–1.72, *p* = 0.049) were independently associated with AGA. In contrast, higher serum 25(OH)D was independently associated with lower odds of AGA (aOR = 0.91 per ng/mL, 95% CI: 0.89–0.94, *p* < 0.001), whereas higher Hcy was independently associated with higher odds of AGA (aOR = 1.48 per μmol/L, 95% CI: 1.37–1.60, *p* < 0.001).

### Model diagnostics and internal validation

Before fitting the multivariable models, collinearity among candidate predictors was assessed using variance inflation factors (VIFs). No substantial multicollinearity was detected (all VIFs <5). Penalized regression yielded generally consistent directions of association, although some effect estimates in smaller subtype-specific models, particularly the U-type model, were attenuated, suggesting limited stability. In addition, penalized regression was used for internal validation. The overall pattern of associations remained broadly consistent; however, the subtype-specific models for smaller BASP subgroups, particularly the U-type subgroup, showed relatively limited stability. Therefore, these subtype-specific findings should be interpreted cautiously.

### Multivariable regression analyses for the severity of different BASP subtypes

To further identify factors independently associated with the severity of each BASP subtype after adjustment for potential confounders, multivariable regression analyses were performed for M-, C-, U-, F-, and V-type AGA (Table 10). Age, father’s hair loss history, high-fat diet, lower serum 25(OH)D levels, and higher Hcy levels were independently associated with severity across all five BASP subtypes.

**Table 10:** Multivariable regression analyses for the severity of different BASP subtypes.

Variables	M-type aOR (95% CI)	*p*-Value	C-type aOR (95% CI)	*p*-Value	U-type aOR (95% CI)	*p*-Value	F-type aOR (95% CI)	*p*-Value	V-type aOR (95% CI)	*p*-Value
Male sex	1.51 (1.16–1.97)	0.002	1.32 (0.95–1.84)	0.098	1.88 (1.04–3.40)	0.036	1.41 (1.08–1.85)	0.012	1.29 (0.91–1.84)	0.152
Age (per year)	1.06 (1.04–1.08)	<0.001	1.05 (1.02–1.08)	<0.001	1.07 (1.02–1.12)	0.004	1.04 (1.02–1.06)	<0.001	1.05 (1.02–1.08)	0.001
Smoking	1.11 (0.90–1.38)	0.332	1.14 (0.82–1.60)	0.432	1.51 (0.93–2.47)	0.096	1.18 (0.91–1.54)	0.205	1.73 (1.18–2.54)	0.005
Drinking	1.02 (0.81–1.28)	0.872	1.02 (0.71–1.46)	0.914	1.69 (1.00–2.86)	0.049	1.34 (1.01–1.80)	0.045	1.71 (1.12–2.61)	0.013
Father’s hair loss history	2.24 (1.76–2.85)	<0.001	2.46 (1.73–3.49)	<0.001	3.10 (1.78–5.40)	<0.001	1.91 (1.44–2.53)	<0.001	1.63 (1.11–2.39)	0.012
Mother’s hair loss history	1.33 (0.96–1.84)	0.083	1.39 (0.90–2.15)	0.138	1.84 (1.01–3.35)	0.047	1.42 (0.98–2.05)	0.066	1.51 (0.94–2.43)	0.089
BMI (per kg/m^2^)	1.03 (1.00–1.07)	0.048	1.04 (1.00–1.09)	0.041	1.06 (1.00–1.13)	0.048	1.03 (0.99–1.07)	0.121	1.04 (0.99–1.10)	0.106
Shampooing interval ≤1 day	1.19 (0.97–1.47)	0.091	1.21 (0.87–1.68)	0.26	1.41 (0.87–2.29)	0.164	1.20 (0.91–1.58)	0.194	1.18 (0.81–1.73)	0.384
Scalp oiliness	1.39 (1.11–1.73)	0.004	1.48 (1.05–2.09)	0.024	1.67 (0.95–2.93)	0.075	1.34 (1.01–1.79)	0.043	1.62 (1.07–2.46)	0.022
High-fat diet	1.74 (1.40–2.17)	<0.001	1.83 (1.30–2.59)	<0.001	2.06 (1.21–3.52)	0.008	1.66 (1.25–2.20)	<0.001	2.14 (1.42–3.22)	<0.001
Hypertension	1.08 (0.79–1.49)	0.621	1.18 (0.77–1.82)	0.442	1.72 (1.01–2.93)	0.045	1.19 (0.84–1.69)	0.329	1.58 (1.01–2.48)	0.046
Diabetes	1.16 (0.71–1.90)	0.554	1.11 (0.51–2.41)	0.795	1.83 (0.90–3.72)	0.093	1.57 (1.01–2.45)	0.046	1.28 (0.63–2.60)	0.496
Heart disease	1.62 (0.83–3.18)	0.159	1.41 (0.39–5.07)	0.6	1.89 (0.52–6.88)	0.335	1.66 (0.64–4.29)	0.297	2.08 (0.73–5.95)	0.17
Hyperlipidemia	1.21 (0.97–1.50)	0.086	1.26 (0.89–1.79)	0.188	1.58 (0.94–2.65)	0.084	1.37 (1.02–1.84)	0.036	1.71 (1.12–2.61)	0.013
Fatty liver	1.29 (0.96–1.74)	0.091	1.31 (0.84–2.06)	0.233	1.82 (1.01–3.30)	0.047	1.29 (0.86–1.94)	0.219	1.44 (0.84–2.47)	0.186
25(OH)D (per ng/mL)	0.93 (0.90–0.96)	<0.001	0.92 (0.88–0.96)	<0.001	0.90 (0.85–0.96)	0.001	0.93 (0.90–0.96)	<0.001	0.91 (0.87–0.96)	<0.001
Hcy (per μmol/L)	1.31 (1.20–1.44)	<0.001	1.28 (1.15–1.42)	<0.001	1.34 (1.15–1.56)	<0.001	1.23 (1.13–1.34)	<0.001	1.27 (1.13–1.42)	<0.001

Adjusted for sex, age, smoking, drinking, father’s hair loss history, mother’s hair loss history, BMI, shampooing interval, scalp oiliness, high-fat diet, hypertension, diabetes, heart disease, hyperlipidemia, fatty liver, serum 25(OH)D, and Hcy.

For M-type AGA, male sex, older age, father’s hair loss history, higher BMI, scalp oiliness, high-fat diet, lower 25(OH)D, and higher Hcy were independently associated with severity. For C-type AGA, older age, father’s hair loss history, scalp oiliness, high-fat diet, lower 25(OH)D, and higher Hcy remained significant after adjustment. For U-type AGA, male sex, older age, drinking, both paternal and maternal hair loss history, higher BMI, high-fat diet, hypertension, fatty liver, lower 25(OH)D, and higher Hcy were independently associated with severity. For F-type AGA, male sex, older age, drinking, father’s hair loss history, scalp oiliness, high-fat diet, diabetes, hyperlipidemia, lower 25(OH)D, and higher Hcy were independently associated with severity. For V-type AGA, older age, smoking, drinking, father’s hair loss history, scalp oiliness, high-fat diet, hypertension, hyperlipidemia, lower 25(OH)D, and higher Hcy were independently associated with severity. Given the multiplicity arising from the five subtype-specific multivariable models, the corresponding associations should be interpreted as exploratory, especially when statistical significance was marginal.

## Discussion

AGA is a common non-scarring alopecia characterized by progressive miniaturization of hair follicles, clinically presenting as gradual hair thinning, reduced hair density, and progressive hair loss. As the disease advances, it may substantially affect appearance, psychological wellbeing, work, and social functioning. Because the onset of AGA is often insidious and its progression is gradual, identifying clinically relevant associated factors may facilitate earlier recognition and more individualized management of affected patients (15, 16). In the present study, we compared patients with AGA and healthy controls and further evaluated factors associated with the severity of different BASP subtypes. Importantly, beyond the initial univariate comparisons, we additionally performed multivariable regression analyses to account for potential confounding among interrelated variables (17). After adjustment, several demographics, familial, lifestyle, and metabolic factors remained independently associated with AGA overall, and both shared and subtype-specific independent associations were identified for the severity of different BASP subtypes. Although additional model diagnostics and internal validation were performed, the subtype-specific findings, particularly those derived from smaller BASP subgroups, should be interpreted cautiously because limited sample size may still affect model stability.

In the multivariable model for overall AGA, male sex, older age, smoking, paternal and maternal history of hair loss, higher BMI, shampooing interval ≤1 day, scalp oiliness, high-fat diet, heart disease, hyperlipidemia, fatty liver, lower serum 25(OH)D, and higher Hcy remained independently associated with AGA. These findings suggest that AGA is associated not with a single exposure but with the combined influence of constitutional predisposition, family history, lifestyle-related factors, and metabolic status. Among these variables, paternal hair loss history showed one of the strongest associations, highlighting the important contribution of inherited susceptibility to AGA. Similarly, the independent associations of lower 25(OH)D and higher Hcy with AGA suggest that metabolic and nutritional status may be relevant in the clinical assessment of patients with this disorder.

When the severity of BASP subtypes was analyzed separately, several factors showed consistent independent associations across all five subtype models. Older age, father’s hair loss history, high-fat diet, lower serum 25(OH)D levels, and higher Hcy levels were independently associated with severity of M-, C-, U-, F-, and V-type AGA. This pattern is noteworthy because it suggests that some variables may represent common correlates of phenotype severity across BASP subtypes. Age likely reflects the cumulative effect of long-term follicular miniaturization and disease duration, whereas paternal hair loss history may reflect a strong genetic contribution. In addition, the consistent associations of high-fat diet, lower vitamin D status, and elevated Hcy with more severe hair loss raise the possibility that metabolic dysregulation, chronic low-grade inflammation, and microvascular impairment may be relevant to the observed associations across BASP phenotypes. However, given the retrospective observational design, these findings should be interpreted as independent associations rather than evidence of causality.

At the same time, our results also suggest meaningful subtype-specific differences. Male sex remained independently associated with severity in M-, U-, and F-type AGA but not in C- or V-type after adjustment. Drinking was independently associated with U-, F-, and V-type severity, whereas smoking remained independently associated only with V-type severity. Hypertension was independently associated with U- and V-type severity, diabetes with F-type severity, hyperlipidemia with F- and V-type severity, and fatty liver with U-type severity. These findings suggest that, although different BASP subtypes share several common associated factors, they may differ in their independent clinical correlates after multivariable adjustment. Such heterogeneity supports the clinical value of BASP classification, not only for describing hair loss patterns but also for exploring differences in associated factors among phenotypes. In the present study, shorter shampooing interval and scalp oiliness were associated with AGA in the overall analysis. However, because these variables were based on self-reported routine clinical assessment rather than objective quantitative measurement, they should be interpreted cautiously.

This study compared AGA patients across different BASP subtypes with various factors. The results indicate that gender, age, smoking, parental history of hair loss, BMI, shampooing frequency, scalp oiliness, high-fat diet, heart disease, hyperlipidemia, fatty liver, 25(OH)D, and Hcy are factors associated with all androgenetic alopecia BASP subtypes. Chronic alcohol consumption is an associated factor for U-type, F-type, and V-type AGA; hypertension is an associated factor for U-type and V-type AGA; and diabetes is an associated factor for U-type and F-type AGA (18). Elevated levels of androgens and dihydrotestosterone (DHT) in males readily interact with hair follicle receptors, triggering a signaling cascade that inhibits follicular growth. This leads to gradual follicular atrophy and ultimately causes AGA (19). Human aging leads to follicular aging, diminishing the differentiation and proliferation capacity of follicular stem cells. This process is accompanied by cumulative DNA damage and alterations in the follicular niche, accelerating the progression of AGA (20, 21). Family history is a well-established major associated factor for AGA. Genetic susceptibility genes increase the sensitivity of androgen receptors within hair follicle cells, enhancing their inhibitory effect on hair growth. This has been hypothesized to be related to follicular miniaturization and the clinical manifestation of AGA (22). Previous studies have proposed several pathways that may explain the observed association between smoking and AGA: It damages microvessels in the dermal papilla, causing local ischemia, and induces increased release of pro-inflammatory factors in anagen hair, leading to follicular microinflammation and fibrosis (23). Alcohol abuse disrupts hormonal and vitamin metabolism, thereby suppressing hair follicle stem cell regeneration and hair growth, promoting the onset and progression of AGA (24, 25). Previous literature has suggested that a high-fat diet may be associated with altered scalp sebum production and follicular microenvironment changes, increases fatty acid synthesis, and disrupts the follicular microenvironment. Concurrently, lipophilic bacteria become active, triggering chronic low-grade scalp inflammation that impairs hair growth. Excess sebum accumulation mechanically compresses hair roots, causing follicular hyper keratinization and plugging, leading to gradual follicular miniaturization (26). Furthermore, prolonged high-fat diets be related to obesity, hypertension, hyperlipidemia, fatty liver disease, cardiovascular disease, and diabetes. These conditions increase blood viscosity, impairing nutrient supply to hair follicles. This results in slowed hair growth and alters growth cycles, exacerbating hair loss (27). AGA patients tend to wash their hair more frequently. This may stem from increased scalp sebum production, where excess oil causes discomfort and prompts more frequent washing. On the other hand, excessive shampooing may disrupt the scalp microbiome, including species such as *Malassezia* and *Propionibacterium acnes*, thereby contributing to AGA pathogenesis (28). 25(OH)D plays a crucial role in preventing hair loss: (1) It regulates keratinocyte differentiation and proliferation, stimulating hair follicle stem cell regeneration and initiating hair growth; (2) 25(OH)D exhibits anti-inflammatory and immunomodulatory functions, helping reduce scalp inflammation and be associated with scalp health; (3) 25(OH)D suppresses androgen levels. Deficiencies in 25(OH)D or its receptor function can lead to loss of hair follicle stem cell function, disruption of the hair growth cycle, elevated androgen levels, increased scalp inflammation, and consequently heightened risk of AGA (29, 30). Hcy is a sulfur-containing amino acid crucial in methionine metabolism and serves as a biomarker for cardiovascular health. It influences scalp blood supply via the circulatory system, thereby affecting nutrient delivery to hair follicles and indirectly increasing AGA risk. Additionally, elevated Hcy levels heighten oxidative stress and inflammatory responses in the body, impairing hair follicle cell activity and proliferation. This can induce alterations in the hair growth cycle and even lead to hair loss (31).

### Clinical implications and future directions

The present findings may have potential relevance for improving the clinical characterization of AGA and for generating hypotheses regarding shared and subtype-specific associated factors across BASP phenotypes. In particular, several demographic, familial, lifestyle, and metabolic variables were independently associated with overall AGA or with severity of specific BASP subtypes. These associations may be useful in informing future risk stratification frameworks and mechanistic studies. However, because the present study was observational, single-center, and non-interventional in nature, the findings should not be interpreted as sufficient evidence to support specific treatment recommendations or personalized management strategies.

Future research should further investigate the biological mechanisms underlying these associations and determine whether the identified factors have prognostic or therapeutic relevance. Multicenter prospective studies and interventional trials are needed to confirm these findings and to clarify whether modification of these associated factors can influence the clinical course of AGA or the response to treatment.

This study has several limitations. First, it was a retrospective single-center case–control study, which may limit generalizability and introduce selection bias. Second, the control group was not individually matched to cases on age, sex, or other baseline variables, and therefore, residual confounding cannot be completely excluded despite multivariable adjustment. Third, some lifestyle-related variables, particularly scalp oiliness and shampooing frequency, were based on self-report and routine clinical history-taking rather than on standardized validated quantitative instruments. Fourth, patients with a previous treatment history for AGA were excluded to reduce treatment-related heterogeneity; however, this may have introduced selection bias by underrepresenting patients with more severe, longstanding, or treatment-resistant disease. Fifth, although BASP classification was independently reviewed and discrepancies were resolved by consensus, a formal inter-rater reliability statistic was not calculated retrospectively. Finally, because of the observational nature of this study, the identified factors should be interpreted as independently associated variables rather than confirmed causal determinants. In addition, given the number of candidate predictors included in the multivariable analyses and the relatively small sample sizes of some BASP subtype groups, particularly the U-type subgroup, overfitting remains a potential concern despite the additional diagnostic procedures performed. Although multicollinearity was assessed using variance inflation factors and penalized regression was applied for internal validation, the stability of some subtype-specific models may still be limited. Therefore, these results should be interpreted as exploratory, especially for smaller BASP subgroups. Although Bonferroni correction was applied to the univariate analyses, no formal multiple-comparison correction was applied across the five subtype-specific multivariable models. Therefore, some subtype-specific associations, particularly those with borderline *p*-values, may represent false-positive findings and should be interpreted cautiously as exploratory. Although multivariable adjustment was performed, the standardized differences indicated substantial baseline imbalance for several variables, and residual confounding cannot be excluded. Some factors showed moderate-to-large imbalance between cases and controls, which should be considered when interpreting the adjusted associations.

## Conclusion

In conclusion, in this retrospective case–control study, several demographics, familial, lifestyle, and metabolic factors were independently associated with AGA overall, and both shared and subtype-specific associations were identified for the severity of different BASP subtypes. Older age, father’s hair loss history, high-fat diet, lower serum 25(OH)D, and higher Hcy were consistently associated with severity across all five BASP subtypes, while several other variables showed subtype-specific associations. These findings may be related to improved clinical assessment and future hypothesis generation in AGA, but prospective studies are still required before these associations can be translated into personalized management strategies or treatment recommendations.

## Data Availability

The original contributions presented in the study are included in the article/Supplementary material, further inquiries can be directed to the corresponding author.
