# Supplementary material for: Clinical characteristics and factors associated with different BASP subtypes of androgenetic alopecia
Source: Front Med (Lausanne). 2026 May 8;13:1757538. doi: 10.3389/fmed.2026.1757538 (PMC13194031; doi:10.3389/fmed.2026.1757538)
Supplement: Supplementary file 1 [file Table_1.DOCX]

Table S1. Baseline characteristics of healthy controls and patients with AGA, with standardized differences

| **Variable** | **Healthy controls (n=1751)** | **AGA cases (n=1505)** | **Standardized difference** |
| --- | --- | --- | --- |
| Male sex, n (%) | 872 (49.80) | 928 (61.66) | 0.241 |
| Age, years, mean ± SD | 27.33 ± 6.29 | 29.86 ± 7.62 | 0.362 |
| Smoking, n (%) | 477 (27.24) | 584 (38.80) | 0.248 |
| Drinking, n (%) | 1108 (63.28) | 934 (62.06) | 0.025 |
| Father’s hair loss history, n (%) | 273 (15.59) | 742 (49.30) | 0.772 |
| Mother’s hair loss history, n (%) | 85 (4.85) | 148 (9.83) | 0.192 |
| BMI, kg/m², mean ± SD | 22.18 ± 4.71 | 23.06 ± 5.23 | 0.177 |
| Shampooing interval ≤1 day, n (%) | 503 (28.73) | 796 (52.89) | 0.507 |
| Scalp oiliness, n (%) | 864 (49.34) | 1037 (68.90) | 0.406 |
| Dandruff, n (%) | 802 (45.80) | 723 (48.04) | 0.045 |
| Sleep duration >8 h, n (%) | 184 (10.51) | 173 (11.50) | 0.032 |
| High-fat diet, n (%) | 468 (26.73) | 893 (59.34) | 0.697 |
| Hypertension, n (%) | 288 (16.45) | 279 (18.54) | 0.055 |
| Diabetes, n (%) | 68 (3.88) | 73 (4.85) | 0.047 |
| Heart disease, n (%) | 5 (0.29) | 26 (1.73) | 0.145 |
| Hyperlipidemia, n (%) | 395 (22.56) | 529 (35.15) | 0.281 |
| Fatty liver, n (%) | 98 (5.60) | 167 (11.10) | 0.2 |
| Serum 25(OH)D, ng/mL, mean ± SD | 31.42 ± 5.36 | 26.65 ± 4.93 | 0.926 |
| Hcy, μmol/L, mean ± SD | 8.32 ± 1.36 | 11.51 ± 1.79 | 2.007 |

Abbreviations: AGA, androgenetic alopecia; BMI, body mass index; Hcy, homocysteine; SD, standard deviation.

Note: Standardized differences are shown as absolute values. For continuous variables, the standardized difference was calculated as the difference in means divided by the pooled standard deviation. For binary variables, it was calculated from the difference in proportions using the pooled binomial variance. Absolute standardized differences of <0.10 were considered negligible, 0.10–0.20 small, 0.20–0.50 moderate, and >0.50 large imbalance.
